# Comprehensive Evaluation of Physicochemical Properties and Microbial Dynamics of Cheka: Traditional Fermented Beverage in Southern Ethiopia

**DOI:** 10.1155/ijm/7912854

**Published:** 2025-10-14

**Authors:** Dawit Albene, Addisu Fekadu Andeta, Kidist Ali, Yonas Syraji, Fitsum Dejene, R. S. Suresh Kumar

**Affiliations:** ^1^Department of Biology, College of Natural and Computational Sciences, Arba Minch University, Arba Minch, Ethiopia; ^2^Department of Chemistry, College of Natural and Computational Sciences, Arba Minch University, Arba Minch, Ethiopia

**Keywords:** Cheka, Ethiopia, fermentation, Konso, Madhota, microbial dynamics

## Abstract

Cheka is a grain- and vegetable-based traditionally fermented beverage usually consumed in southwest Ethiopia, mainly in Konso and Derashe. Conventionally, it is made by spontaneous fermentation and therefore mostly relies on the actions of autochthonous microorganisms. Due to the lack of scientifically documented information on the microbiology of the Cheka fermentation process, we undertook this study to explore the microbial profiles and physicochemical properties involved in Cheka fermentation. Cheka is traditionally prepared in the laboratory using aseptic procedures with locally sourced ingredients from Arba Minch. The physicochemical properties and dominant and spoilage organisms at various stages of fermentation and in the final products were analyzed. The pH reduced from 4.10 to 2.88, while the titratable acidity changed from 0.06% to 1.60% lactic acid during 0 to 48 h of fermentation. Protein content increased from 1.57 ± 0.04 to 3.62 ± 0.09 and from 1.67 ± 0.08 to 3.91 ± 0.06 at 0 h and 48 h for Cheka and Madhota. A significant increase in carbohydrate content was observed in Cheka and Madhota from 21.14 ± 1.95 to 51.51 ± 3.00 g/100 g and from 12.11 ± 1.95 to 51.58 ± 2.95 g/100 g, respectively. The analysis of microbial counts in Cheka showed that the total viable count (TVC) increased from 8.72 to 9.4 log during 0–48 h of fermentation. Yeast and mold counts increased from 6.71 to 8.80 log (CFU/mL). *Clostridium* was removed within 48 h of fermentation, and *Enterobacteriaceae* showed a significant (*p* < 0.05) reduction from 4.90 to 1.77 ± 0.20 and from 4.60 ± 0.30 to 1.34 log (CFU/mL) in both Cheka and Madhota products. The alcohol content of Cheka increased from 3.00% to 16.00% (*v*/*v*) between 0 and 48 h of fermentation. The findings of this study provide a comprehensive understanding of the relationship between physicochemical parameters and the microbial characteristics of Cheka during fermentation. This understanding will aid in ensuring the safe production and enhancing the quality of Cheka and similar products in the future.

## 1. Background

Fermented beverages have been produced and consumed worldwide for a long time and are thought to have begun approximately 6000 bc [1, 2]. These beverages are indigenous to a particular area and have been developed using age-old techniques by native people utilizing the locally available raw materials. These fermented products play a crucial role in the diets of people worldwide, serving as both food and drink [3]. Indigenous people prefer fermented foods because they can be preserved for long periods, and the presence of diverse microorganisms enhances the sensory qualities, nutritional value, digestibility, aroma, texture, and shelf life of the product, making them ideal and pleasant for consumption at social events [4]. Many types of fermented beverages are consumed in Africa for a variety of social events, such as marriage, rain-making ceremonies, festivals, social gatherings, and funerals. Many fermented products prepared by people involve the use of household components and are produced using methods that adhere to cultural systems [5].

Ethiopia produces approximately 8 million hL of traditionally fermented alcoholic beverages annually [6]. In Ethiopia, as in other parts of the world, a significant amount of traditionally fermented foods and beverages are produced using locally available grains and vegetables, either combined or used separately [5]. These are known by various names, including Cheka, Tej, Tella, Borde, Shameta, Keribo, Korefe, Imbushbush, Bukire, and Merissa [5, 7]. Cheka is a fermented food and drink commonly consumed in the southwestern regions of Ethiopia, particularly in Dirashe and Konso. Cheka is not only popular in the southwestern parts of Ethiopia but also consumed in other regions such as South Omo, Gamo, Walayta, Sidama, Burjji, Amarokelle, Alle, Gujji, Yabello, Gedieo, Hadiya, and Basketo due to its high energy-producing metabolic qualities, which are ideal for farming and tasks that require more physical labor [8, 9]. Cheka, made from sorghum, maize, malt, and water, is a valuable food source for low-income people as it is a cheap and nutritious meal alternative.

Depending on the presence or absence of malt and the methods of preparation, there are four types of Cheka produced in Ethiopia, especially in the Konso Region. These are Cheka, Shurkuta, Errorota (Errera), and Madhota (pulla) [8]. In the cases of Cheka and Errorota, malt served as a catalyst. Unlike Shurkuta, Madhota does not contain malt and is predominantly used by Protestant Christians due to its low or negligible alcohol content compared to Cheka and Errorota [8]. When it comes to the preparation method, both Cheka and Madhota are prepared in a similar way with three major phases of fermentation, except for the use of malt in the case of Cheka [9].

Microorganisms are key drivers of fermentation, and the presence of specific microbial communities in both the environment and raw materials can significantly impact the final product [9]. Lactic acid bacteria and yeasts are essential microorganisms in traditional fermented foods [10]. The fermentation processes and microbiological dynamics of Ethiopian fermented foods and beverages, such as Tella, Borde, and Shamita, have been studied previously [11, 12]. Cheka is now widely available throughout southern Ethiopia because of its popularity, easily accessible ingredients, and affordability. Despite the high volume of production and consumption of Cheka, its fermentation process remains spontaneous, unregulated, and unpredictable [13], with no scientifically documented information available. To enhance the quality of Cheka and facilitate large-scale production, a deeper understanding of its physicochemical properties and microbial composition during fermentation is necessary. Therefore, this study is aimed at exploring the physicochemical properties of fermented Cheka and its microbial dynamics during the entire fermentation process.

## 2. Materials and Methods

### 2.1. Description of the Study Area

**Figure 1:**
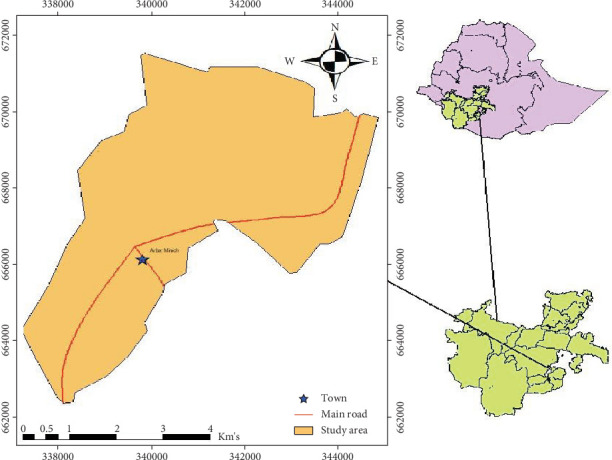
Location map of the study area.

The study was conducted at Arba Minch University, Arba Minch, Gamo Zone, in the southwestern part of Ethiopia. Arba Minch town is indeed located at 6°2⁣^″^ N latitude and 37°33⁣^″^ E longitude, approximately 500 km south of Addis Ababa and at an elevation of 1285 m (Figure 1). The current estimate of the total population in the zone (permanent residents) is approximately 115,639 residents [14]. Its total area has been estimated to be 10,000 km^2^, lying at an elevation of 710 to 4200 m above sea level. The Gamo zone is subdivided into districts (woredas), and kebeles are the smallest administrative units within the districts. The average temperature and annual rainfall in the Arba Minch town are 10°C–25°C and 200–2000 mm, respectively [15].

### 2.2. Collection of Ingredients and Preparation of Cheka in the Laboratory

**Figure 2:**
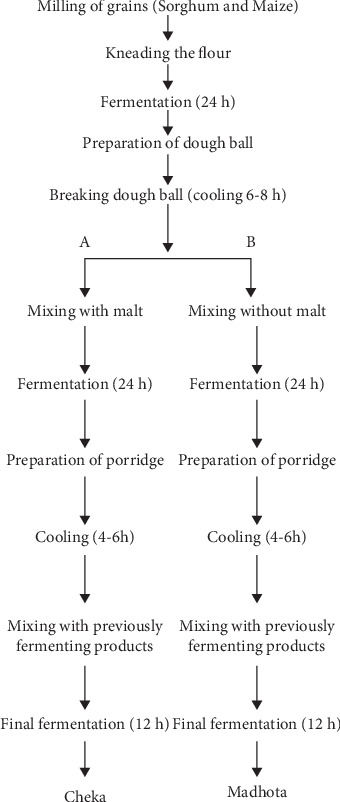
The overall Cheka and Madhota preparation procedures.

The ingredients used to prepare Cheka were collected from local markets in Arba Minch Town. The ingredients used for preparing Cheka in this study were maize flour, red sorghum malt made from red sorghum, and water. For this work, two kinds of fermented products, Cheka (with malt) and Madhota (without malt), were prepared in the laboratory according to the traditional procedure followed by the native Konso people of Southern Ethiopia. Aseptic techniques were adopted during preparation to avoid any contamination. As indicated in Figure 2, Cheka undergoes three fermentation phases. The first phase involved milling maize (3 kg) and red sorghum (3 kg), followed by kneading with water by mixing equal amounts (1:1) of the flour and allowing it to ferment for 24 h. Kurkufa or dough balls were created after a 24-h fermentation period. The dough balls were then cooked to gelatinize the cereal starch granules and enhance the ability of the amylase enzyme to break down starch. The cooked dough balls were then broken and allowed to cool for 6–8 h. In the second phase, the broken dough balls were further made into small pieces and kneaded with malt (one-fourth of the total amount of Maize and Sorghum flour used for Cheka) and without malt (for Madhota) in two different sterile plastic jars as fermenters. The mixture was allowed to ferment for 24 h at room temperature. After 24 h of fermentation, a small amount of flour (maize or sorghum) was mixed with boiling water to form a semiliquid material (porridge) and allowed to cool to room temperature. The cooled porridge was then mixed with a small amount of malt to initiate fermentation, locally known as Absit. In the third phase, the prepared porridge was mixed with the fermented products from the second phase and allowed to ferment for 12 h at room temperature to obtain pure Cheka. The final product was allowed to ferment for a prolonged period (0–48 h), and this period was determined based on the preference of the local people, who usually know the best time to consume the final fermented product. Physicochemical and microbial profiling were performed after obtaining the final product. Fermentation was performed in a 500-mL sterile plastic sealed with parafilm. A single batch of the fermentation process was carried out, but the products were allowed to run for a set time, from 0 to 48 h. A destructive sampling technique was used each time, meaning that once the jar was opened, it was not used for the second time to prevent cross-contamination. The fermentation process for Madhota was similar to that of Cheka, except that no malt was added to Madhota.

### 2.3. Determinations of Physicochemical Parameters of Cheka Sample

During the Cheka and Madhota fermentation, samples were taken every 4 h on the first day and every 12 h on the second and third days for analysis. These samples were tested for pH, acidity, and microbial changes. The pH of fermenting samples was measured by homogenizing 1 mL for 60 s in 9 mL of distilled water in a stomacher (Star BlenderTM 145 133 LB 400, VWR International, Fontenay Sous Bois Cedex, 134, France) and measured at 0, 4, 8, 12, 24, and 48 h of fermentation as described by Assaye et al. [16]. Then, the pH of the samples was recorded by dipping the glass electrode of a digital pH meter into 10 mL of the sample. At each fermenting time, the temperature in the fermenting sample was monitored by entering a disinfected digital thermometer (Traceable Long-Stem 140 Thermometers, Stainless steel probe, Bestone Meter Ltd, Shenzhen, China) at the surface, middle, and bottom in each fermenting time, and the readings were recorded in relation to room temperature condition according to Assaye et al. [16]. Titratable acidity (TA) was measured using a method described by Assaye et al. [16]. Briefly, TA samples were determined by taking approximately 1 g of each of the fermenting samples and mixing them in 9 mL of distilled water using a stomacher bag. The supernatant was measured directly by titrating 10 mL. Then, it was determined by titrating with 0.1 M NaOH, using 1% phenolphthalein as an indicator. As the color of the sample changed from clear to pink, the volume of NaOH consumed until the endpoint was reached was used to calculate the TA. The moisture content of the samples was determined according to the official method of the AOAC [15]. Briefly, the drying dish was dried in an oven at 105°C for 1 h and placed in desiccators to cool. The weight of the drying dish was determined and denoted as (*W*1). Five grams of the samples was weighed in the dry dish (*W*2) and oven dried at 105°C for 24 h, and after cooling in a desiccator to room temperature, it was again weighed (*W*3):

 $$\begin{array}{c} \text{Total moisture }\left(\%\right)=\frac{W1-W2}{W1\;}*100, \end{array}$$

where *W*1 is the weight of the fresh sample and *W*2 is the weight of the dried sample.

### 2.4. Proximate Composition Analysis

#### 2.4.1. Determination of Ash Content

The ash content was measured according to the dry ashing procedure using the official method of the AOAC (2000). The crucible was then raised to room temperature in a desiccator for approximately 30 min, before being weighed on a digital balance (*W*1). Five grams of the freeze-dried Cheka sample was weighed and placed in a clean crucible (*W*2). It was then burned on a hot plate at a low temperature until the smoke died down to prevent spattering. The sample was incinerated at 550°C for 5 h in a muffle furnace and then burned until it turned white. The total ash content was determined using the equation given below:

 $$\begin{array}{c} \%\text{Total ash}=\frac{W3‐W1\;}{W2‐W1}*100, \end{array}$$

where *W*1 is the weight of the crucible, *W*2 is the weight of the crucible + sample, *W*3 is the weight of the crucible + ash, *W*2‐*W*1 is the weight of the sample, and *W*3‐*W*1 is the weight of the ash.

#### 2.4.2. Determination of Crude Fiber

The crude fiber content was analyzed according to the AOAC guidelines [15]. About 2 g (*W*3) of the sample was transferred into a 400-mL beaker. After digestion with 1.25% sulfuric acid, the sample was washed with distilled water, digested with 1.25% NaOH, and then filtered. The residue left after refluxing, washed again with 1.25% sulfuric acid near the boiling point. The residue was then dried at 95°C overnight, cooled in desiccators, and weighed (*W*1). After mashing for 2 h at 500°C, it was cooled in desiccators and weighed again (*W*2). The weighted sample (*W*2) was cooled in a desiccator for 30 min after being burned for approximately 30 min at 300°C in a muffle furnace and then reweighed (*W*3). The crude fiber content was calculated by the equation below:

 $$\begin{array}{c} \%\text{Crude fiber}=\frac{\text{W}2-\text{W}3}{\text{W}1\;}*100, \end{array}$$

where *W*1 is the weight of the sample, *W*2 is the crucible weight after ashing, and *W*3 is the crucible weight after drying.

#### 2.4.3. Determination of Crude Fat

The determination of crude fat in the sample was carried out using the Soxhlet extraction method with diethyl ether according to the official method of the AOAC [15]. Approximately 5 g of the freeze-dried sample was weighed in a thimble containing fat-free cotton (*W*2), and 150 mL of petroleum ether was added to the flask fitted with the Soxhlet extraction apparatus. This procedure began with a known quantity of the sample (*w*1) in a round-bottomed flask containing a few antibumping granules. The round-bottom flask was then dried after being cleaned with distilled water and detergent. The flask was weighed (*W*3) after cooling in a desiccator for 30 min. The solvent was then evaporated by heating in a steam bath. The flask containing the extracted fat was dried in a steam bath to a constant mass. The total crude fat was calculated as a percentage by weight:

 $$\begin{array}{c} \text{Crude fat},\text{percent by weight}=\frac{W2-W3}{w1\;}*100, \end{array}$$

where *W*1 is the weight of the sample, *W*2 is the weight of the flask, *W*3 is the weight of the flask with fat, and *W*3‐*W*2 is the weight of fat.

#### 2.4.4. Determination of Crude Protein

The protein content of the samples was determined using the Kjeldahl method, according to the official method of the AOAC [15]. Two grams of traditionally fermented Cheka was digested by adding 5 mL of concentrated sulfuric acid in the presence of a potassium sulfate catalyst in a Kjeldahl flask. The digest was then neutralized and distilled, and the resulting digest was diluted with 30 mL of distilled water. Then, 25 mL of NaOH (40%) was added to neutralize the sulfuric acid. The liberated ammonia was collected in 30 mL of 1% boric acid solution containing a mixed indicator. Finally, the ammonium attached to the borate anion was titrated with standardized HCl, and the total crude protein was calculated as total nitrogen.

#### 2.4.5. Determination of Total Carbohydrate

The total carbohydrate content was determined according to the AOAC [15] by difference: Carbohydrate content was determined by subtracting the values of the other components from 100. Total carbohydrate (%) = 100 − (%*M* + %*P* + %*F* + %FI + %*A*), where *M* is the moisture and *P* is the crude protein. The energy value was calculated using the Atwater conversion factors.

### 2.5. Determination of Ethanol Content in Cheka Sample

The ethanol content in Cheka and Madhota was determined using a UV/Vis spectrophotometer at an appropriate wavelength, as described by [17]. Quantification was performed by preparing a series of standard solutions of absolute alcohol and measuring their absorbance at 590 nm. Five grams of the samples was measured and mixed with 30 mL of distilled water and vortexed. The mixture was centrifuged at 5000 rpm for 15 min and filtered through Whatman No. 1 to obtain a clear supernatant. The ethanol content of the supernatant was determined from the measured absorbance using a standard calibration curve.

### 2.6. Determinations of Microbial Dynamics During Cheka Fermentation

To count the number of microbes, 5 g of each Chaka sample was mixed with 45 mL of a salt solution containing peptone (0.85% NaCl and 0.1% peptone, Biokar Diagnostics, Beauvais, France). The mixture was then blended for 60 s using a homogenizer (Star BlenderTM LB 400, VWR International, France). A subsequent serial dilution was done and pour-plated in sterile plates using the pour plate technique, according to the ISO principles described by Asasye et al. [16]. The total number of viable aerobic bacteria was counted on plates containing plate count agar (PCA) (Biokar Diagnostics) after incubation at 30°C for 72 h. To count the number of lactic acid bacteria (LAB), the samples were plated on de Man, Rogosa, and Sharpe (MRS, Biokar Diagnostics) medium, and the grown colonies were counted after incubation at 30°C for 72 h. The presence of Enterobacteriaceae in Cheka samples was evaluated using violet red bile glucose agar (VRBG) (Biokar Diagnostics) after incubation at 37°C for 24 h. The total yeast and mold counts were determined on oxytetracycline glucose agar (OGA) (Biokar Diagnostics) supplemented with oxytetracycline, and the growth potential of the colony was counted after incubation at 25°C for 3–5 days. Finally, the plates were incubated at 37°C for 48 h in anaerobic jars containing indicator strips and gas-generating kits [16].

### 2.7. Mineral Analysis of Fermented Cheka

The mineral composition of the samples was determined according to the methods recommended by the Association of Official Analytical Chemists and the ASEAN Manual of Food Analysis (ASEAN FOODS, 2011). The levels of sodium, potassium, calcium, magnesium, copper, iron, and zinc were measured using atomic absorption spectrophotometry (AA-7000 Series, Shimadzu Scientific Instruments, Kyoto, Japan) following standard procedures [15].

### 2.8. Growth Potential of Certain Pathogens in Fermented Cheka

Cheka samples were individually tested against standard test strains obtained from the Ethiopian Public Health Institute (EPHI) to determine which pathogenic organisms could survive during prolonged fermentation when acidity and other organic acid production increased. The strains used in this study included *Staphylococcus aureus* (ATCC25923), *Listeria monocytogenes* (ATCC19115), *Escherichia coli* (ATCC25922), and *Pseudomonas aeruginosa* (ATCC27853). Later, 200 mL of the Cheka sample was steamed at 80°C for 10 min to destroy any somatic cells that might be found in the prepared Cheka sample. Then, from the steamed sample, 50 mL was tested separately with an overnight refreshed 0.5 mL culture of the test strains to make the final inoculum level 10^3^–10^4^ CFU/g. Serial dilutions were prepared by homogenizing 1 mL of the steamed sample in 9 mL of sterile saline solution (0.85% *w*/*v* NaCl). Then, the pour plate was done from an appropriate dilution by taking 1 mL of eosin methylene blue agar for *E. coli*, mannitol salt agar for *S. aureus*, *Listeria* selective agar for *L. monocytogenes*, and LB agar for *P. aeruginosa*. A portion of the beverage sample was further sampled aseptically at time intervals from 0 to 48 h, as described by Andeta et al. [18], with slight modifications, and the counts were expressed as log (CFU/g). A steamed Cheka sample without inoculation of pathogenic strains was used as a negative control.

### 2.9. Statistical Analysis

For statistical analysis, triplicate determinations were made. Data are presented as mean ± standard deviation of two independent calculations. One-way ANOVA was used to analyze the variations in the physicochemical, proximate composition, and microbiological counts of the chosen samples over the course of the various time periods. Tukey's HSD was employed to identify significant differences in total solids, crude fat, crude protein, crude fiber, total ash, total carbohydrate, and gross energy content of Cheka using a significance level of (*p* < 0.05).

## 3. Result and Discussion

### 3.1. Determinations of Physicochemical Parameters in Cheka Sample

**Figure 3:**
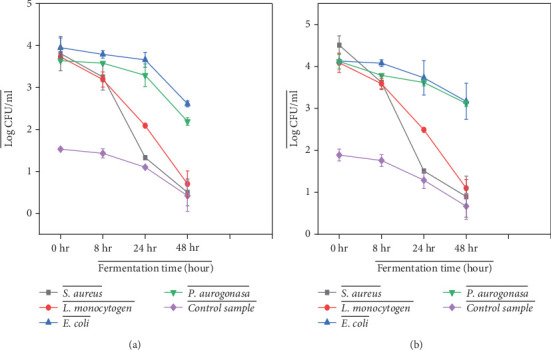
Risk assessment of pathogenic bacteria in steamed Cheka log (CFU/g) ((a) for Cheka and (b) for Madhota).

The pH profile and total TA of Cheka at different fermentation times are presented in Figure 3. The pH of the Cheka samples decreased significantly (*p* < 0.05) with fermentation time. The pH of the sample decreased from 4.10 ± 0.06 to 2.90 ± 0.02 in Cheka and from 3.85 ± 0.17 to 3.33 ± 0.03 in Madhota at 0–48 h, respectively, as indicated in Table 1. This significant reduction in pH observed in both *Cheka* and *Madhota* over the 48-h fermentation period might indicate active microbial fermentation, especially by LAB and yeasts.

This acidification serves not only to enhance the safety and shelf life of the beverage by inhibiting pathogenic and spoilage organisms but also plays a key role in developing its characteristic sour taste. Freire et al. [19] found that the pH of the fermented product decreased significantly (*p* < 0.05) from 6.45 ± 0.05 to 3.65 ± 0.01 as fermentation time increased from 0 to 48 h. Another study by Nemo and Bacha [20] indicated that the pH value showed a significant decrease from 4.5 to 3.0 when the fermentation time increased from 0 to 60 h. The internal temperature during the fermentation of Cheka varied, as shown in Table 1. The highest mean temperature (25.05°C) was observed at the eighth hour of fermentation, and the lowest mean value (16.25°C) was observed at the 24th hour of fermentation, while the room temperature at the eighth hour was 24°C and 24th hour was 23°C, respectively. As we observed, an increase in internal temperature during *Cheka* fermentation is primarily attributed to internal microbial metabolic activity at a certain period of time and showed variation, although environmental temperature may contribute slightly since fermentation is a known exothermic process. In a related study by Assaye et al. [16], when the fermentation time increased, the internal temperature decreased. Similarly, Abawari [3] discovered that the internal temperature was reduced from 22.67 ± 0 41 to 21.96 ± 0 23 during the fermentation time from 0 to 24 h. The other important physicochemical test was TA. TA increased from 0.06 ± 0.00 to 1.25 ± 0.03 and from 0.07 ± 0.00 to 1.56 ± 0.06 during the fermentation time of 0–48 h. As fermentation time increased, the level of total TA also increased. This is likely because the LABs produce organic acids. When compared with Cheka, Madhota had the highest increase in TA level (1.56 ± 0.06) compared to Cheka (1.25 ± 0.03). A related study by Anyiam et al. [21] reported that TA increased from 2.14 ± 0.01 to 3.24 ± 0.24 mL NaOH/100 g, while pH reduced from 4.8 ± 0.05 to 3.25 ± 0.03 during 0–30 days of fermentation. According to Rebouças et al. [22], the TA among three different maturity days of Tej (Ethiopian traditional fermented beverage) varied from 0.42 ± 0.11 to 1.16 ± 0.35. Moisture content decreased significantly (*p* < 0.05) during fermentation from 73.42 ± 0.38 to 42.26 ± 0.05 and from 82.45 ± 0.19 to 42.11 ± 0.34 for Cheka and Madhota, respectively. The present study is in line with Freire et al. [19], who observed a reduction (*p* < 0.05) in moisture when the fermentation time increased.

### 3.2. Proximate Composition of Cheka Sample

The amount of ash, moisture, crude fat, crude fiber, protein, and carbohydrate in food is important for determining its nutritional value and quality. In this study, proximate parameters such as ash, fat, fiber, protein, and total carbohydrate were determined, and the results are shown in Table 2. The data in the table indicate that the ash content in the samples increased with increasing fermentation time. The total ash content increased from 0.56 ± 0.01 to 0.74 ± 0.04 in Cheka and from 0.59 ± 0.07 to 0.73 ± 0.02 in Madhota, but the changes were not significant (*p* > 0.05). An increase in protein content was observed from 1.57 ± 0.04 and 1.67 ± 0.08 at 0 h to 3.62 ± 0.09 and 3.91 ± 0.06 at 48 h for both Cheka and Madhota, respectively. This increase in protein content may be attributed to the accumulation of microbial biomass, microbial protein synthesis, and also a reduction in antinutritional compounds that may bind and interfere with the entire protein during detection. According to Nemo and Bacha [20], a significant increase in protein content was observed during the maturation of Yakupa, along with a decrease in pH. Freire et al. [19] reported a similar study, which showed a reduction (*p* < 0.05) in crude fiber and carbohydrate content and an increase in ash and protein content with an increase in fermentation time. A significant reduction in crude fat content (from 1.56 ± 0.09 to 1.15 ± 0.16 and from 1.41 ± 0.17 to 0.99 ± 0.08 g/100 g) and a decrease in crude fiber content (from 1.74 ± 0.12 to 0.71 ± 0.09 and from 1.77 ± 0.13 to 0.70 ± 0.09 g/100 g) for Cheka and Madhota were observed with an increase in fermentation time from 0 to 48 h. The observed decrease in crude fat and fiber might be due to the production of lipase enzyme, which degrades triglyceride into fatty acid and glycerol, and the enzymatic degradation of complex structural carbohydrates by microbial enzymes.

According to Nemo and Bacha [20], fat content decreased from 51.9 ± 1.9 to 1.3 ± 0.1 g L^−1^ during Yakupa fermentation from 0 to 60 h. A significant increase in carbohydrate content was observed from 21.14 ± 1.95 to 51.51 ± 3.00 g/100 g and from 12.11 ± 1.95 to 51.58 ± 2.95 g/100 g for Cheka and Madhota, respectively. This may be due to the type and amount of ingredients used and the role of microorganisms. In a related study by Tsegaye et al. [13], the mean total carbohydrate content (g/100 g) of Cheka samples was found to be 59.11. In the present study, the gross energy (Kcal/100 g) content ranged from 111.87 ± 1.90 to 233.73 ± 2.99 and from 74.91 ± 2.66 to 233.62 ± 1.10 for Cheka and Madhota, respectively. The current study is related to that of Tsegaye et al. [13], where the gross energy content of Cheka samples ranged from 270.79 to 287.87.

### 3.3. Effect of Fermentation Time on Alcoholic Contents of Cheka

In this study, the alcoholic content of Cheka increased from 3.33 ± 0.03 to 16.00 ± 0.03, and that of Madhota increased from 3.00 ± 0.30% to 12.60 ± 0.30% *v*/*v* during 0–48 h of fermentation, as shown in Table 1. Compared with Cheka, Madhota had a significantly (*p* < 0.05) lower alcoholic content, which might be due to the absence of malt in Madhota. In this study, the alcohol content in Cheka was found to be higher than that of Berhanu et al. (3.35% and 5.44% *v*/*v*, respectively) [23]. This result indicates that initially, the level of ethanol in Cheka was similar to that of Tella (2.5%–14.52%) and Tej (6.2%–14% *v*/*v*) but increased as fermentation time increased. According to Kemal and Koricha [5], the alcohol content increased from 5.22 ± 0.74 to 14.1 in Jikita fermentation (traditional fermented beverage popular among the Oromo ethnic groups in Ethiopia) from 0 to 24 h. The ethanol content increased from 10.07 ± 0.71 to 12.00 ± 0.85 for another well-known Ethiopian fermented beverage, Tej, when the maturation time increased [22]. Similarly, in our study, the different set of Cheka samples had a higher alcohol concentration than those found in the investigations by Wedajo [24] and Tsegaye et al. [13]. This is most likely due to the increase in fermentation time, which may have led to an increase in alcohol content. A similar study reported by Nemo and Bacha [20] showed that the ethanol content increased when the fermentation time increased from 0 to 60 h.

### 3.4. Effect of Fermentation Time on the Microbial Count During the Cheka Fermentation Process

The quality of fermented foods is influenced by changes in the microbial population during fermentation. The microbial dynamics of Cheka, including LAB, TVC, Enterobacteriaceae, *Clostridium*, yeast, and mold, were evaluated, and the results are presented in Table 3. As seen from the table, the number of LAB in Cheka and Madhota increased from 7.20 to 8.41 log (CFU/g) in Cheka and from 7.10 to 8.13 log (CFU/g) in Madhota until 24 h of fermentation. After 24 h of fermentation, LAB showed some reduction as compared to TVC, yeast, and mold counts. The TVC is the total viable count of microbes, and in this case, the count increased from 8.32 to 9.40 log (CFU/g) in the case of Cheka and from 8.16 to 8.99 log (CFU/g) in the case of Madhota, as shown in Table 3.

The other most important bacterial group considered in this work was *Enterobacteriaceae*, which showed a significant decrease from 4.90 to 1.77 log (CFU/g) and from 4.60 to 1.34 log (CFU/g) when the fermentation time increases from 0 to 48 h in Cheka and Madhota, respectively. These are pathogenic bacteria that cannot survive when the pH is below 4.0. The decrease in *Enterobacteriaceae* count may be due to the lowering of the pH during the fermentation process by LAB. Another significant bacterium identified in the fermented products was the aerobic spore-forming *Clostridium*. The *Clostridium* count decreased from 3.90 to <1.00 log (CFU/g) and 2.95 to <1.00 log (CFU/g) in Cheka and Madhota, respectively. Significant differences (*p* < 0.05) were observed when the fermentation hour increases from 0 to 48 h, and the counts were below the detection limit of 1 log (CFU/g) [25, 26]. This could be due to the highly acidic stress conditions formed by LAB and yeasts, which are not favorable for them.

As reported by Binitu et al. [27], the TVC count increased from the mean value of 7.04 to 8.29 log (CFU/g) when the maturation time increased. According to Assaye et al. [16], when fermentation time increased, *Enterobacteriaceae* declined to below the detection level (<1.00 log [CFU/g]). The total yeast and mold counts were also determined in this study, as they are directly associated with food fermentation. The yeast and mold counts increased from 6.81 to 8.80 log (CFU/g) and from 6.71 to 8.52 log (CFU/g) in Cheka and Madhota, respectively. Since the pH of the system decreased, this may be preferable for yeasts and molds [22].

### 3.5. Effect of Fermentation Time on Mineral Composition of Cheka

The basic minerals found in the Cheka and Madhota samples were also present in Cheka. The effect of fermentation time on the mineral content of Cheka is presented in Table 4. The findings showed a substantial increase in mineral content (*p* < 0.05) corresponding to an increase in maturation time. The Cheka sample showed the greatest increment in Fe (26.43 ± 0.50) and Ca (24.81 ± 0.12) after 48 h of fermentation. This may be due to the fact that LAB and other fermenting microbes produce organic acids and reduce antinutritional factors by enhancing mineral bioavailability.

The mineral compositions were found to be Fe > Ca > Mg > Zn > K > Cu > Na in the overall fermentation process of Cheka and Madhota from 0 to 48 h. Madhota has less mineral content than Cheka, which could be because Madhota contains no malt, which contributes to less fermentation.

The results showed that Cheka had a high iron level, which might be explained by the fact that red sorghum is typically used to prepare Cheka, which could be the source of the increased iron content in Cheka. A similar investigation was reported by Tsegaye et al. [13], who found that the Fe content in Cheka's beverage ranged from 11.70 to 0.05 mg/L. Wedajo and Getnet and Berhanu [24, 28] revealed that the Ca and Fe contents in Cheka samples taken from various locations in southwest Ethiopia ranged from 8.31% to 19.6% and 14.41% to 18.96%, respectively. Furthermore, an increase in maturation time from 0 to 48 h may have caused the content variation. Tsegaye et al. [13] reported that the average zinc concentration in Cheka samples obtained from various sources ranged from 2.48 to 5.33 mg/L. A related study by Freire et al. [19] found that as fermentation time increases from 0 to 42 h, the amounts of minerals such as Fe, Zn, and Ca increased. The increase in mineral content may be due to the type of ingredients used, and it may also be contributed to by the water used during the preparation of the fermented product.

### 3.6. Growth Potential of Certain Pathogens in the Fermented Beverages

In fermented products, it is essential to understand the potential growth of pathogens to ensure safety and quality [18]. Steaming fermented foods and drinks at 80°C for 10 min can help prevent spoilage, improve safety, and extend the shelf life of Cheka and Madhota samples. The growth potential of the test pathogens in the Cheka and Madhota samples was tested separately using standard test strains, and a reduction in the growth potential of pathogenic bacteria was observed (Figure 3). In this case, a statistically significant (*p* < 0.05) reduction was observed for *E. coli* (ATCC25922) from 3.95 ± 0.23 to 2.61 ± 0.09 and *S. aureus* (ATCC25923) from 3.81 ± 0.41 to <1 log (CFU/g). The reduction in the growth of potential pathogens during fermentation may be due to the production of antimicrobial substances such as lactic acid. Compared with gram-positive bacteria, gram-negative bacteria showed resistance in both samples when the fermentation time was increased from 0 to 48 h. This might be because gram-negative bacteria have an outer membrane made of lipopolysaccharides (LPSs), which acts as an additional protective barrier and resistance to tolerant stress conditions. The current study showed that steamed Cheka and Madhota samples had relatively lower pathogen content than other Ethiopian fermented foods, as previously reported for Borde and Korefe fermented products [18, 29]. The production of organic acids, lowering of pH, and production of alcohols during fermentation play crucial roles in the inhibition of pathogenic bacterial growth.

There were no significant differences in the growth of the test strains on the control sample compared to the inoculated sample (*p* < 0.05). The bacterial count in the control sample was much lower than that in the inoculated sample, as shown in Figure 3. This is due to the fact that the control sample did not contain any pathogenic strains and only contained naturally occurring or background microflora, which decreased when fermentation time increased.

## 4. Conclusion

This study provides comprehensive insights into the physicochemical characteristics and microbial dynamics of Cheka and Madhota, traditional fermented beverages widely consumed in Southern Ethiopia. The results showed progressive changes in pH, TA, temperature, and other key physicochemical parameters across different fermentation times from 0 to 48 h. This study also revealed a significant decrease in spoilage microorganisms, such as *Enterobacteriaceae* and *Clostridium*, when the fermentation time increased. This study underscores the importance of fermentation time as a critical factor influencing both the microbial diversity and physicochemical properties of *Cheka*, which can help in standardization efforts for quality control and improving the health benefits. Therefore, it is recommended to consume Cheka fresh to obtain better gross energy and to abstain from high alcohol content. To unlock the full potential of *Cheka* as a culturally valuable and microbiologically safe functional beverage, further research is required to elucidate the total microorganisms up to the species level using advanced metagenomic tools and to conduct controlled fermentation trials to scale up the production of this fermented beverage.

## Figures and Tables

**Table 1:** Results of physicochemical parameters of fermented Cheka and Madhota samples.

**Parameters**	**Sample**	**M** **e** **a** **n** ± **SD**	**M** **e** **a** **n** ± **SD**	**M** **e** **a** **n** ± **SD**	**M** **e** **a** **n** ± **SD**	**M** **e** **a** **n** ± **SD**	**M** **e** **a** **n** ± **SD**
		**0** h	**4** h	**8** h	**12** h	**24** h	**48** h
Tem (°C)	Cheka	23.50 ± 0.50^a^	24.40 ± 0.00^a^	25.05 ± 0.20^a^	17.55 ± 0.00^b^	16.25 ± 0.05^a^	22.85 ± 0.02^a^
	Madhota	22.95 ± 0.2^a^	24.10 ± 0.06^b^	24.45 ± 0.00^b^	17.08 ± 0.02^a^	18.55 ± 0.01^a^	22.65 ± 0.03^a^

pH	Cheka	4.06 ± 0.06^b^	3.90 ± 0.01^a^	3.66 ± 0.04^a^	3.38 ± 0.00^a^	3.19 ± 0.05^a^	2.90 ± 0.02^a^
	Madhota	3.85 ± 0.17^b^	3.68 ± 0.02^a^	3.56 ± 0.02^a^	3.41 ± 0.12^a^	3.38 ± 0.10^a^	3.33 ± 0.02^a^

TA	Cheka	0.06 ± 0.00^a^	0.06 ± 0.01^a^	0.10 ± 0.02^a^	1.11 ± 0.01^a^	1.13 ± 0.02^a^	1.25 ± 0.03^a^
	Madhota	0.07 ± 0.00^a^	0.07 ± 0.00^a^	1.13 ± 0.00^a^	1.35 ± 0.04^a^	1.37 ± 0.01^a^	1.56 ± 0.09^b^

% Moisture	Cheka	73.42 ± 3.29^a^	71.42 ± 1.57^a^	68.11 ± 0.98^b^	61.25 ± 5.03^b^	54.72 ± 4.97^a^	42.26 ± 4.27^a^
	Madhota	82.45 ± 0.65^a^	69.44 ± 0.32^a^	64.90 ± 4.96^b^	61.27 ± 8.11^a^	61.20 ± 11.10^a^	42.11 ± 3.94^a^

*Note:* Small letter (a) in a row indicates significant difference (*p* < 0.05), while (b) indicates no significant difference (*p* < 0.05).

Abbreviations: TA: titratable acidity; Temp: temperature.

**Table 2:** Results of proximate analysis.

**Parameters**	**Sample**	**M** **e** **a** **n** ± **SD**	**M** **e** **a** **n** ± **SD**	**M** **e** **a** **n** ± **SD**	**M** **e** **a** **n** ± **SD**	**M** **e** **a** **n** ± **SD**	**M** **e** **a** **n** ± **SD**
		**0** h	**4** h	**8** h	**12** h	**24** h	**48** h
% Ash	Cheka	0.56 ± 0.01^a^	0.61 ± 0.04^a^	0.69 ± 0.07^a^	0.83 ± 0.13^a^	0.59 ± 0.04^a^	0.74 ± 0.04^a^
	Madhota	0.59 ± 0.07^a^	0.60 ± 0.03^a^	0.66 ± 0.00^b^	0.77 ± 0.09^a^	0.79 ± 0.01^a^	0.73 ± 0.02^b^

% Protein	Cheka	1.57 ± 0.04^a^	1.77 ± 0.03^a^	1.83 ± 0.16^a^	2.60 ± 0.16^a^	3.12 ± 0.13^a^	3.62 ± 0.09^a^
	Madhota	1.67 ± 0.08^a^	1.78 ± 0.10^a^	1.93 ± 0.05^a^	2.80 ± 0.12^a^	3.46 ± 0.15^a^	3.91 ± 0.06^a^

% Crude fiber	Cheka	1.74 ± 0.12^a^	1.66 ± 0.11^a^	1.54 ± 0.04^a^	1.51 ± 0.05^b^	1.37 ± 0.03^a^	0.71 ± 0.09^a^
	Madhota	1.77 ± 0.13^a^	1.63 ± 0.02^a^	1.51 ± 0.04^b^	1.50 ± 0.04^b^	1.31 ± 0.16^a^	0.70 ± 0.09^a^

% Crude fat	Cheka	1.56 ± 0.09^a^	1.52 ± 0.09^a^	1.50 ± 0.09^b^	1.39 ± 0.13^b^	1.27 ± 0.13^b^	1.15 ± 0.16^a^
	Madhota	1.41 ± 0.17^a^	1.33 ± 0.08^a^	1.30 ± 0.08^a^	1.19 ± 0.06^a^	1.10 ± 0.05^a^	0.99 ± 0.08^a^

% Carbohydrate	Cheka	21.14 ± 1.95^a^	23.03 ± 2.95^a^	26.36 ± 1.95^a^	32.43 ± 2.9^a^	38.93 ± 2.42^b^	51.51 ± 3.00^a^
	Madhota	12.11 ± 1.95^a^	25.23 ± 2.95^a^	29.72 ± 1.95^a^	32.47 ± 1.95^b^	32.15 ± 1.95^b^	51.58 ± 2.95^a^

Gross energy (Kcal)	Cheka	111.87 ± 1.90^a^	119.50 ± 2.61^a^	132.05 ± 2.47^a^	158.58 ± 2.10^a^	185.11 ± 2.95^a^	233.73 ± 2.99^a^
	Madhota	74.91 ± 2.66^a^	126.47 ± 2.10^a^	144.10 ± 2.95^a^	157.76 ± 2.10^a^	157.45 ± 1.95^a^	233.62 ± 1.10^a^

%Alcoholic content (%*v*/*v*)	Cheka	3.33 ± 0.30^a^	3.50 ± 0.30^a^	3.73 ± 0.35^a^	5.52 ± 0.20^b^	8.70 ± 0.30^a^	16.00 ± 0.30^a^
	Madhota	3.00 ± 0.30^a^	3.15 ± 0.30^a^	3.68 ± 0.30^a^	4.03 ± 0.35^b^	5.75 ± 0.20^a^	12.60 ± 0.30^a^

*Note:* Small letter (a) in a row indicates significant difference (*p* < 0.05), while (b) indicates no significant difference (*p* < 0.05).

**Table 3:** Microbial counts log (CFU/g) throughout the fermentation times.

**Sample**	**Microbial group**	**0** h	**4 h**	**8** h	**12 h**	**24** h	**48** h
Cheka	LAB	7.20 ± 0.30^a^	7.61 ± 0.20^a^	8.10 ± 0.30^a^	8.22 ± 0.20^a^	8.41 ± 0.30^a^	7.74 ± 0.30^b^
Madhota	LAB	7.10 ± 0.30^a^	7.30 ± 0.30^a^	7.93 ± 0.20^a^	8.01 ± 0.20^a^	8.13 ± 0.30^a^	7.34 ± 0.30^a^
Cheka	TVC	8.32 ± 0.03^a^	8.46 ± 0.20^a^	8.75 ± 0.20^b^	8.79 ± 0.20^b^	9.12 ± 0.30^a^	9.40 ± 0.30^a^
Madhota	TVC	8.16 ± 0.03^a^	8.40 ± 0.30^a^	8.68 ± 0.20^b^	8.90 ± 0.20^a^	8.96 ± 0.20^a^	8.99 ± 0.20^a^
Cheka	Entro	4.90 ± 0.30^a^	4.70 ± 0.20^a^	4.41 ± 0.20^a^	3.73 ± 0.20^b^	3.23 ± 0.20^a^	1.77 ± 0.20^a^
Madhota	Entro	4.60 ± 0.30^a^	4.35 ± 0.15^a^	4.10 ± 0.29^a^	3.36 ± 0.30^b^	2.91 ± 0.20^a^	1.34 ± 0.30^a^
Cheka	YAM	6.81 ± 0.20^a^	6.95 ± 0.20^a^	6.97 ± 0.30^a^	7.18 ± 0.20^b^	8.33 ± 0.30^a^	8.80 ± 0.20^a^
Madhota	YAM	6.71 ± 0.20^a^	6.83 ± 0.20^a^	6.87 ± 0.20^a^	7.01 ± 0.20^b^	7.70 ± 0.30^a^	8.52 ± 0.30^a^
Cheka	Clost	3.90 ± 0.30^a^	3.31 ± 0.20^a^	3.20 ± 0.20^a^	2.81 ± 0.15^a^	1.23 ± 0.30^a^	<1.00 ± 0.20^a^
Madhota	Clost	2.95 ± 0.20^a^	2.90 ± 0.20^a^	2.64 ± 0.20^b^	2.51 ± 0.20^b^	1.14 ± 0.20^a^	<1 ± 0.20^a^

*Note:* Small letter (a) in a row indicates significant difference (*p* < 0.05), while (b) indicates no significant difference (*p* < 0.05).

Abbreviations: Clost: Clostridium; Entro: Enterobacteriaceae; LAB: lactic acid bacteria; TVC: total viable count; YAM: yeast and mold.

**Table 4:** Mineral contents in the Cheka fermentation process.

**Fermentation time**	**Sample**	**Na (mg/L)**	**K (mg/L)**	**Ca (mg/L)**	**Mg (mg/L)**	**Cu (mg/L)**	**Fe (mg/L)**	**Zn (mg/L)**
0 h	Cheka	1.18 ± 0.10^a^	2.62 ± 0.25^a^	12.05 ± 0.20^a^	9.22 ± 0.20^a^	1.14 ± 0.01^a^	12.38 ± 0.30^a^	5.24 ± 0.20^a^
	Madhota	0.80 ± 0.20^a^	1.53 ± 0.20^a^	8.79 ± 0.20^a^	8.35 ± 0.30^a^	1.11 ± 0.10^a^	10.71 ± 0.20^a^	2.57 ± 0.30^a^

8 h	Cheka	1.52 ± 0.20^b^	2.88 ± 0.10^a^	12.57 ± 0.20^b^	12.45 ± 0.20^a^	1.98 ± 0.03^a^	13.48 ± 0.40^b^	6.14 ± 0.10^b^
	Madhota	1.10 ± 0.10^a^	1.86 ± 0.20^a^	9.98 ± 0.32^a^	10.70 ± 0.20^a^	1.66 ± 0.20^a^	7.64 ± 0.20^a^	3.43 ± 0.40^a^

24 h	Cheka	2.98 ± 0.10^a^	3.38 ± 4.10^a^	16.12 ± 0.20^a^	13.18 ± 0.10^a^	2.68 ± 0.20^a^	19.81 ± 2.10^b^	8.36 ± 0.30^b^
	Madhota	2.12 ± 0.10^a^	3.12 ± 0.10^a^	11.70 ± 0.20^a^	11.13 ± 0.10^b^	2.21 ± 0.20^b^	11.70 ± 0.20^b^	5.09 ± 0.04^b^

48 h	Cheka	3.43 ± 0.40^a^	4.54 ± 0.30^a^	24.81 ± 0.10^a^	16.35 ± 0.30^a^	3.66 ± 0.20^a^	26.43 ± 0.40^a^	9.85 ± 0.10^a^
	Madhota	2.66 ± 0.20^a^	3.46 ± 0.20^a^	15.57 ± 0.20^a^	14.23 ± 0.20^a^	3.44 ± 0.02^a^	18.23 ± 0.20^a^	7.43 ± 0.20^a^

*Note:* Small letter (a) in a row indicates significant difference (*p* < 0.05), while (b) indicates no significant difference (*p* < 0.05).

## Data Availability

All data and materials are mentioned in this paper.

## Nomenclature

LAB: lactic acid bacteria
TVC: total viable count
CFU/g: colony-forming unit per gram
ANOVA: analysis of variance
PCA: plate count agar
